# Genetic modulation of diabetic nephropathy among mouse strains with Ins2 *Akita* mutation

**DOI:** 10.14814/phy2.12208

**Published:** 2014-11-26

**Authors:** Xiuju Wu, Richard C. Davis, Timothy S. McMillen, Valerie Schaeffer, Zhiqiang Zhou, Hongxiu Qi, Parisa N. Mazandarani, Roshanak Alialy, Kelly L. Hudkins, Aldons J. Lusis, Renée C. LeBoeuf

**Affiliations:** 1 Division of Cardiology, Department of Medicine, David Geffen School of Medicine, University of California, Los Angeles, Los Angeles, California, USA; 2 Division of Metabolism, Endocrinology and Nutrition, Department of Medicine, University of Washington, Seattle, Washington, USA; 3 Department of Pathology, University of Washington, Seattle, Washington, USA; 4 Department of Human Genetics, University of California, Los Angeles, Los Angeles, California, USA

**Keywords:** Akita, Animal models, Albuminuria, Diabetic nephropathy, Genetics

## Abstract

Diabetic nephropathy (DN) is a major complication of diabetes and the leading cause of end‐stage renal disease. DN is characterized by changes in kidney structure and function but the underlying genetic and molecular factors are poorly understood. We used a mouse diversity panel to explore the genetic basis of DN traits in mice carrying the Ins2 Akita mutation. Twenty‐eight Akita strains were generated by breeding this panel to DBA/2.Akita mice. Male F1 diabetic and nondiabetic littermates were evaluated for DN‐related traits. Urine albumin‐to‐creatinine ratios (ACRs), volume and cystatin C as well as blood urea nitrogen and lipoprotein levels varied significantly among the diabetic strains. For most Akita strains, ACR values increased 2‐ to 6‐fold over euglycemic control values. However, six strains exhibited changes in ACR exceeding 10‐fold with two strains (NOD/ShiLt and CBA) showing 50‐ to 83‐ fold increases. These increases are larger than previously reported among available DN mouse models establishing these strains as useful for additional studies of renal function. ACRs correlated with cystatin C (*P* = 0.0286), a measure of hyperfiltration and an interstitial tubular marker associated with DN onset in humans suggesting that tubule damage as well as podocyte‐stress contributed to reduced kidney function assessed by ACR. Although large changes were seen for ACRs, severe nephropathology was absent. However, glomerular hypertrophy and collagen IV content were found to vary significantly among strains suggesting a genetic basis for early onset features of DN. Our results define the range of DN phenotypes that occur among common inbred strains of mice.

## Abstract (precis)

Diabetic nephropathy (DN) is characterized by changes in kidney structure and function but the underlying genetic and molecular factors are poorly understood. We used a mouse diversity panel to explore the genetic basis of DN traits in mice carrying the Ins2 Akita mutation. Twenty‐eight Akita strains on different genetic backgrounds were evaluated for DN‐related traits and the results define the range of DN phenotypes that occur among common inbred strains of mice.

## Introduction

Diabetic nephropathy (DN) is the leading cause of end‐stage renal disease (ESRD) (USRDS: the United States Renal Data System 2003) and is also an independent risk factor for cardiovascular disease in diabetic patients (Sarnak et al. 2003). Among patients with diabetes (type 1 and type 2) the lifetime risk of DN is 20–30% (Rossing et al. 1995; USRDS: the United States Renal Data System 2003). Early manifestations of DN include loss of podocyte density and modestly elevated levels of urine albumin (microalbuminuria) followed 5–10 years later by severe proteinuria (macroalbuminuria) (Fioretto et al. 1992; Pagtalunan et al. 1997; Mauer and Drummond 2002). The major hallmarks of severe DN include glomerular hypertrophy, thickening of the glomerular basement membrane, glomerulosclerosis, mesangial matrix expansion, podocyte loss and tubulointerstitial fibrosis (Fioretto et al. 1992; Arora and Singh 2013). Patients with type 1 diabetes mellitus (T1D) often exhibit enlarged kidneys and elevations in glomerular filtration rate (GFR) (Wiseman et al. 1985; Mauer and Drummond 2002). DN may result in ESRD, particularly in T1D patients, with treatments limited to dialysis or kidney transplantation. Clinical trials focusing on standard therapy to control hyperglycemia and hypertension have not shown markedly improved outcomes (Himmelfarb and Tuttle 2013). In fact, treatment of T1D patient with drugs inhibiting the renin‐angiotensin‐aldosterone system showed no improvement in glomerular pathology although there was delay of overall renal functional impairment (Mauer et al. 2009). Thus, the identification of early markers of DN as well as new therapeutic targets for treatments of DN associated with diabetes remains paramount.

The precise molecular factors involved in DN are poorly understood, and human genetic studies have been only modestly helpful (Arora and Singh 2013). Pathways implicated in the pathogenesis of DN are oxidative stress, protein kinase C, activation of receptor of AGEs and nuclear receptors including the nuclear factor–kB transcription factor family and the JAK/STAT pathway (Schmid et al. 2006; King 2008; Arora and Singh 2013). Specific candidate genes have been derived for DN from association studies and include inflammatory molecules, TGF‐beta receptors, VEGF, angiotensin‐converting enzyme, BMPs, BMP antagonists, and lipid transport genes (reviewed in Brennan et al. 2013). Novel candidate genes have also been identified such as FRMD3 (4.1 protein ezrin, radixin, moesin [FERM] domain containing 3) and CARS (cysteinyl‐tRNA synthatase) in association studies involving a large cohort of T1D and control patients (Pezzolesi et al. 2009). Genome‐wide association studies (GWAS) focusing on T1D DN have been less informative but data support several novel candidate genes including AFF3 and ERBB4 as well as genome‐wide significant QTLs located on other chromosomes (Fagerholm et al. 2012; Sandholm et al. 2012). Roles for these proteins in DN have not been defined or studied in animal models, and prognostic and therapeutic avenues involving these genes have not been developed. Thus, molecular pathways specific for DN remain unclear and the identification of genes influencing susceptibility and resistance to DN would aid in a better understanding of processes leading to DN.

The heterogeneity and variable time of onset of DN have complicated both molecular and genetic studies in humans. Mouse models such as Akita and leptin receptor and ligand deficient mice offer the opportunity to study DN under controlled genetic and environmental conditions. As yet, these mutations have been examined only on a small set of genetic backgrounds in the mouse. In this study, we set out to explore the range of traits associated with DN that occur in mice carrying the Akita mutation.

Because genetic background has a profound effect on DN pathogenesis, we tested the hypothesis that studies of DN across a greater number of inbred mouse strains would provide broader knowledge of DN in mouse models. Further, this information will eventually be used in a systems biology approach to identify genes and gene pathways controlling DN in the mouse system in order to guide future clinical studies toward improved therapy (Bennett et al. 2012; Ghazalpour et al. 2012). Here, we used a large‐scale mouse diversity panel to explore the genetic basis of early onset and progression of DN in mice carrying the *Akita* mutation. Male mice of strain DBA/2 heterozygous for the *Akita* gene were bred to 28 different inbred strains chosen from a panel of inbred strains that we have previously shown to be powerful in association mapping of complex traits (Bennett et al. 2010). Kidney traits were evaluated for diabetic and nondiabetic F1 littermates. We found that mouse strains varied widely in extent of albuminuria (assessed as albumin‐to‐creatinine ratio [ACR]) as well as urine levels of cystatin C (cystatin C–to‐creatinine ratio [CCR]), an interstitial tubular marker associated with DN onset in humans. A robust correlation between ACR and CCR was found suggesting that both traits provide early markers for potential DN. Finally, glomerular hypertrophy was seen for many mouse strains although the extent of this trait did not correlate with ACR. Overall, this report provides a wider record of DN traits across mouse strains as well as provides an important tool for further identification of genes modulating DN.

## Materials and Methods

### Mice and experimental design

Male DBA/2 mice heterozygous for the *Ins2* Akita mutation (DBA.Akita) were purchased from The Jackson Laboratory (Bar Harbor, ME) and bred to females from 28 inbred strains (also purchased from The Jackson Laboratory) in order to generate F1 mice. In the text and most Figures, strain names are listed with respect to female strain and without the “J” (except for strain A/J) denoting the commercial source (The Jackson Laboratory).

Male F1 mice carrying the *Ins2* Akita mutation as well as F1 male euglycemic littermate controls were maintained on pelleted rodent chow (PicoLab rodent Diet 20 #5053; 4.5% fat by weight; 20% protein). Animals were housed in pathogen‐free conditions, in a temperature and humidity‐controlled environment (12‐h light/dark cycle). Mice were weaned at 21 days and housed as 3–5 mice per cage. At 6 weeks of age, mice were assessed for blood glucose (taken via tail‐nick) to identify which mice were diabetic versus euglycemic. Confirmation of genotype was done as described (http://jaxmice.jax.org/protocolsdb/f?p=116:2:::NO:2:P2_MASTER_PROTOCOL_ID,P2_JRS_CODE:176,003548). Mice were maintained for 4–5 months with no insulin treatment at which time they were euthanized by cervical dislocation following isofluorane anesthesia. Blood and urine samples were taken as described below. Tissues were weighed and either snap‐frozen in liquid nitrogen followed by storage at −80°C or placed in buffered formalin for histological and morphological analysis. All animal procedures were reviewed and approved by the Institutional Care and Use Committees at the University of California, Los Angeles and the University of Washington, Seattle.

### Blood and plasma assays

Blood was collected from mice that were fasted for 4–5 h and bled 4 h after the beginning of the light cycle from the retro‐orbital plexus using isofluorane anesthesia immediately prior to euthanasia. Blood glucose levels were monitored using a portable glucose measuring device (AlphaTRAK^®^, Abbott Laboratories, North Chicago, IL). Plasma was isolated by centrifugation in BD Microtainer tubes with EDTA (Becton, Dickinson and Company, Franklin Lakes, NJ). Plasma glucose levels were measured using the glucose oxidase reaction as monitored in a Beckman Glucose Analyzer 2 (Beckman Instruments). Plasma total cholesterol, HDL cholesterol, free cholesterol, triglycerides, and free fatty acid (FFA) concentrations were determined by enzymatic assays employing colorimetric endpoints as described previously (Hedrick et al. 1993; Mehrabian et al. 1993; Puppione and Charugundla 1994). Plasma BUN levels were assessed with a colorimetric assay (Kit #DIUR‐500, BioAssay Systems, Hayward, CA).

### Urine assays

During the week prior to euthanasia, mouse urines were collected and volumes assessed over 24 h using metabolic cages designed for efficient recovery and separation of urine from feces in individual mice (Tecniplast, West Chester, PA). In some cases, spot urines were collected over a period of 4–12 h using a custom‐made mouse urine collection station. The mice had free access to water and food during the time of urine collection. Urine albumin measurements were performed using the Albuwell M ELISA kit (Exocell, Philadelphia, PA) and creatinine levels in the urine were determined by The Creatinine Companion kit (Exocell) according to the manufacturer's instructions. Prior to assays, urines were diluted such that albumin and creatinine levels were in the linear range. Urinary albumin‐to‐creatinine ratios (ACRs) were calculated as microgram urinary albumin per mg urinary creatinine. Quantification of urine cystatin C protein levels were performed by Elisa procedures as described by the manufacturers (#RD291009200R, BioVendor Research, Asheville, NC and #1019, Exocell Inc.). Urine dilutions were 1:20 for cystatin C. For nehprin, Elisa procedures were performed following urine dilutions of 1:10 (Kit #1019, Exocell Inc.). Values were normalized to urine creatinine levels as obtained from the albumin‐to‐creatinine assay.

### Immunohistochemistry and morphometric analysis

Formalin fixed paraffin embedded kidneys were cut into 5 *μ*m sections. For glomerular hypertrophy, one section per kidney per mouse (*n* = 3–4 mice/strain) were stained with H&E. All glomeruli within one complete kidney section were counted and areas determined, excluding the Bowman's capsule. Areas were quantified using ImageJ (NIH) Software (http://rsb.info.nih.gov/ij/) by an observer blinded to animal genotype. Visual inspection of tubule areas were carried out to assess gross damage as a function of diabetes.

For immuno‐staining, sections were deparaffinized, rehydrated, and soaked overnight in water at 65°C. Sections were then treated with protease XXIV (#HK053‐5K, BioGenex, Fremont, CA), followed by routine immunostaining using a kit (Kit #CTS008, R&D Systems, Minneapolis, MN). The antibody to collagen IV was used at a 1:1000 dilution for 1 h at room temperature (#1340‐01, Southern Biotech, Birmingham, AL). An antibody against mouse Ki67 (Abcam, Cambridge, MA) was used to show cellular proliferation. Macrophages were detected using anti‐mouse F4/80 antibody (Abcam). Control sections were generated that had no treatment with primary antibody. Collagen IV staining was quantified within glomeruli and expressed as percent positive immunostaining area per total glomerulus area. Five high‐powered (10X) independent visual fields were imaged from one section per mouse, and data obtained for ≥25 glomeruli (*n* = 3–4 mice/strain). Positive staining was quantified using ImageJ (NIH) software by an observer blinded to animal genotype.

### Statistical analysis

Data are reported as mean ± SEMs and statistical significance was established at *P* < 0.05. Nonparametric ANOVA (Bonferroni correction) analyses followed by multiple comparisons were performed as appropriate, focusing on genotype and diabetic status. Correlations among quantitative traits were done using BiWeight Midcorrelation within the WGCNA package available in R‐language programming (http://www.inside-r.org/packages/cran/WGCNA/docs/bicor) (Langfelder and Horvath 2012). This is an alternative to using Pearson correlations as it is less adversely affected by outlier values.

## Results

Our goal was to access DN across a wide range of mouse genetic backgrounds in order to provide new mouse models of DN and to identify key genetic factors modulating DN severity. We took advantage of the fact that heterozygosity of the *Ins2* Akita mutation causes severe hyperglycemia to generate a panel of F1 mice across a large number of mouse genetic backgrounds. Among the available congenic strains carrying the Akita mutation, the DBA/2 background develops the most severe kidney disease (Brosius et al. 2009; Gurley et al. 2010; Chang and Gurley 2012) and thus, we chose it as the “donor” strain. Female Akita mice tend to be more resistant to the effects of the Akita mutation than male mice exhibiting variable glucose levels. Therefore, we restricted our study to males.

### Albuminuria varied markedly across 28 strains of diabetic mice

It is known that genetic background influences the extent of albuminuria in diabetic patients and mice and that microalbuminura is a risk factor for progression to DN (Long et al. 2013). Thus, we explored the genetic variation in DN by evaluating urine albumin and creatinine concentrations taken from mice at 5 months of age across 28 inbred strains.

**Figure 1.**
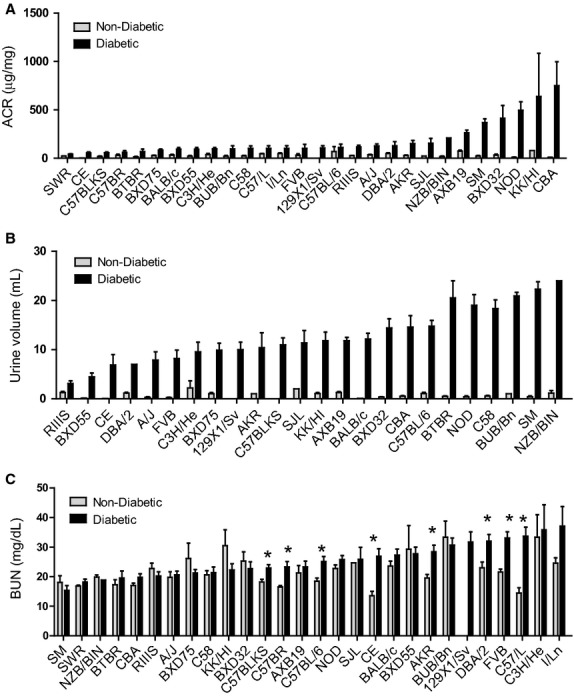
Albuminuria, polyuria and blood urea nitrogen for diabetic and nondiabetic control mice across a panel of 28 F1 mouse strains. To test the impact of genetic background on traits associated with DN, DBA/2.Akita male mice were bred to females of 28 different inbred lines and the resulting male F1 animals were evaluated. F1 animals inheriting the Akita mutation are labeled as diabetic while offspring without the mutation are used as normoglycemic controls. The variation in genetic background as determined by the maternal strain is indicated on the horizontal axis for each graph. For each phenotype, strains are shown in rank order according to strain‐values for that phenotype in diabetic mice. Gray bars show control mice and black bars show diabetic mice. Error bars indicate SEM. (A) Albumin‐to‐creatinine ratios (ACRs, μg urinary albumin per mg urinary creatinine) varied widely among the strains and were elevated for diabetic versus nondiabetic controls in most cases (*n* = 4–17 mice/group). Nearly all strains showed significant increases in ACR with diabetes (*P* < 0.001–0.050). (B) Average urine volume (mL per 24 h). Severe polyuria is seen for diabetic as compared to nondiabetic mice. (C) Blood urea nitrogen (BUN) levels (mg/dL). *P < 0.05, *n* = 4–17. To facilitate comparisons among strains, these data are presented in alphabetical strain order in Figure 7‐1A, 1B and 1C at the end of the manuscript.

Albumin‐to‐creatinine ratios (ACR) were calculated for diabetic mice and nondiabetic controls (Fig. 1A). Among the nondiabetic mice, ACRs varied 80‐fold with KK/HI showing the highest levels. Among diabetic mice, ACR values varied widely with mean ACRs ranging from 42 to 750 *μ*g/mg (Fig. 1A). Strains KK/Hl, CBA and NOD/ShiLt were highly sensitive to the early development and progression of diabetic nephropathy with robust increases in ACR for diabetic versus nondiabetic controls. For instance, ACRs for diabetic CBA and NOD/ShiLt increased 83‐fold and 50‐fold, respectively, over nondiabetic controls. These increases are greater than the suggested increases in ACR of 10‐fold set as a standard to validate, in part, a progressive mouse model of DN (Brosius et al. 2009). Thus, these strains can be considered potential models for DN. More moderate changes in ACR were seen for strains SM (17‐fold), BXD32/Ty (13‐fold), and AXB19/Pgn (4‐fold). Strains SWR, C57BLKS, C57BR/cd, BTBR, BXD75/Rww, C57L, BXD55/Rww, C3H/He, BUB/Bn, and BALB/c were relatively resistant to albuminuria showing modest increases for diabetic mice (2‐ to 5‐fold).

**Figure 2.**
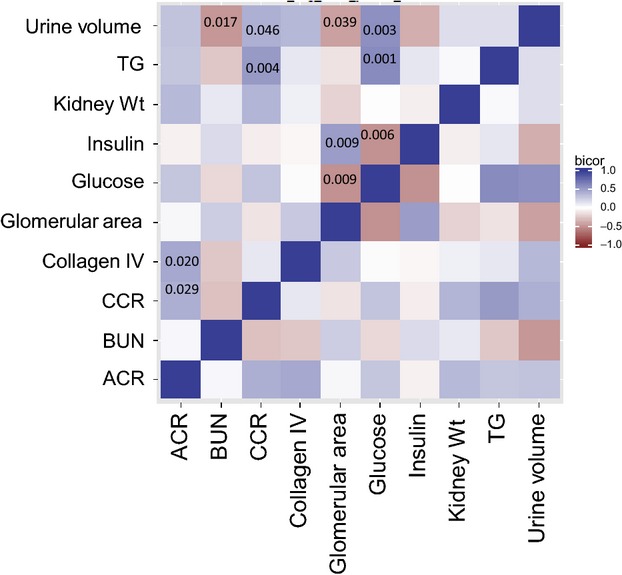
Correlation of nephropathy‐related phenotypes among diabetic mice across a panel of 28 F1 mouse strains using BiWeight Midcorrelation (Langfelder and Horvath 2012). Color indicates positive (blue) or negative (red) correlation (Vlasakova et al. 2014). Intensity of color indicates strength of correlation as shown on the scale. For statistically significant correlations, *P*‐value is given within the color square.

Of note were the marked differences in the extent of polyuria across the Akita strains (Fig. 1B). For nondiabetic mice, values ranged 20‐fold (0.10 mL/day for BALB/c and CE to 2.20 mL/day for C3H/He) and increased robustly and significantly with diabetes for each strain. Daily urine volumes ranged 8‐fold (3.1 mL/day for RIIIS to 24 mL/day for NZB/BIN). ACR and polyuria traits did not correlate significantly across the strains (Fig. 2). For instance, BUB/Bn showed severe polyuria but reduced ACR levels as compared to NOD/ShiLt that had both polyuria and elevated ACR.

Blood urea nitrogen (BUN) levels are also a conventional assessment of renal function. Among the F1 mice, BUN values for diabetic mice ranged from 15 to 37 mg/dL and five strains showed significant elevations in BUN values as compared to nondiabetic counterparts (Fig. 1C). Diabetic BUN values did not correlate significantly with ACRs (Fig. 2).

### Metabolic traits did not correlate with ACR levels

**Figure 3.**
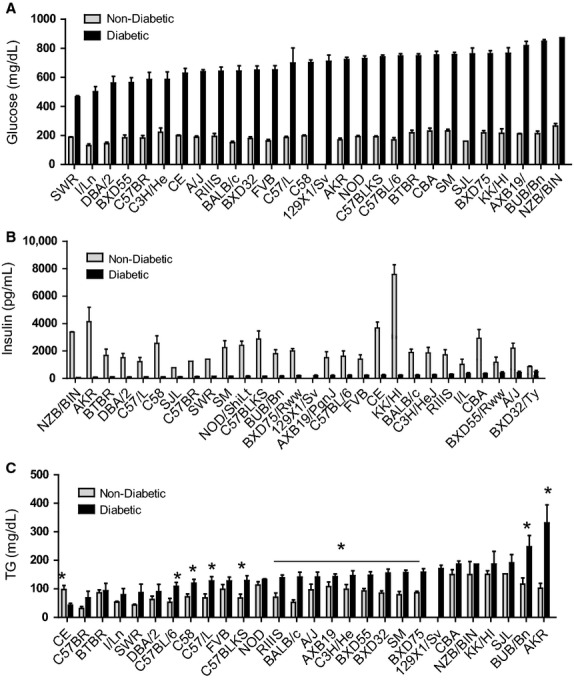
Plasma glucose, insulin, and triglyceride (TG) levels for diabetic and nondiabetic F1 males from a cross between DBA Akita males and a panel of females from various inbred strains. For each graph, the maternal strain is indicated on the horizontal axis and strains are in rank order based on the strain‐average level observed in diabetic mice. (A) Impact of genetic background on plasma glucose levels (mg/dL) is relatively modest among mice of the 28 strains carrying the Akita mutation. There is less than a 2‐fold difference across all strains. (B) As expected, plasma insulin levels (pg/mL) are markedly suppressed in all diabetic F1 males compared to nondiabetic F1 males derived from the same maternal strain. Moreover, insulin levels in diabetic mice were unrelated to levels seen in nondiabetic controls for the same strain. (C) Impact of genetic background on plasma TG levels (mg/dL). *P < 0.05 (*n* = 3–17 mice/group). To facilitate comparisons among strains, these data are presented in alphabetical strain order in Figure 7‐3A, 3B and 3C at the end of the manuscript.

The Akita mutation causes beta‐cell failure as a result of protein aggregate‐induced endoplasmic reticulum stress due to improper folding of proinsulin resulting in reduced insulin production and hyperglycemia (Ron 2002). As expected, Akita mice exhibited severe hyperglycemia at necropsy (~5 months of age) with plasma glucose levels ranging from 465 to 873 mg/dL as compared to values for nondiabetic mice (129 to 264 mg/dL) (Fig. 3A). Of note was that across all of the diabetic strains studied here, there was no significant correlation between plasma glucose levels and ACR (Fig. 2).

Plasma insulin levels varied among the mouse strains (Fig. 3B). For nondiabetic mice, 4‐h fasting levels varied 5‐fold from 782 to 3676 pg/mL. For diabetic mice, insulin levels were markedly reduced and ranged from 60 to 485 pg/mL and correlated negatively with plasma glucose levels (Fig. 2; *P* = 0.006).

Because diabetes is often associated with alterations in lipid levels (Bogdanovic 2008), total plasma cholesterol (TC), triglyceride (TG), and high density lipoprotein (HDL) levels were evaluated across the 28 strains. TG levels (Fig. 3C) for most strains were significantly elevated for diabetic versus nondiabetic mice and for diabetic mice, were significantly correlated to plasma glucose levels (Fig. 2; *P* = 0.001). Plasma TC levels remained comparable between diabetic and nondiabetic cohorts except for a few strains for which TC was significantly reduced (C3H/He, FVB, KK/Hl) or elevated by diabetes (C58, AKR). Variable responses to diabetes were seen among the 28 strains for HDL levels with many strains showing decreases, increases and no change with diabetes. No correlations were seen between TC or HDL and ACR values (data not shown) suggesting that cholesterol levels are not a direct indicator of early DN among these strains.

Body weights of diabetic mice were ~25% lower than those of nondiabetic controls (data not shown) except for four strains where body weights were comparable for control and Akita mice (BxD32, BxD55, I/Ln, and SWR strains). Nonetheless, mice looked healthy based on coat condition and activity levels despite the severe hyperglycemia.

**Figure 4.**
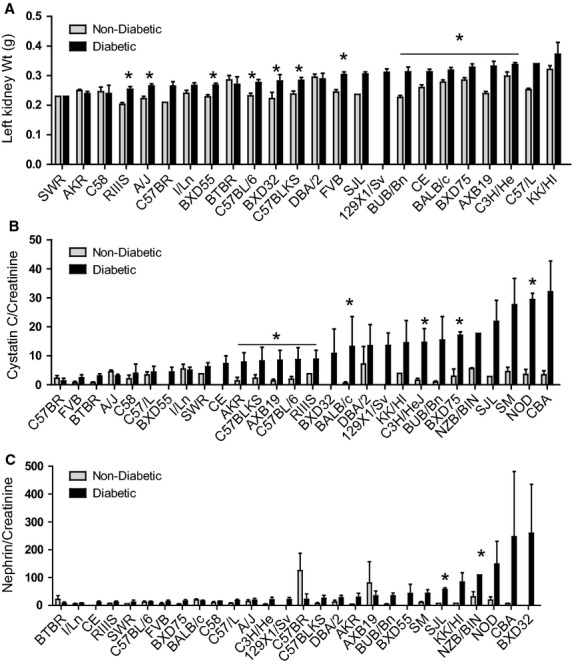
Impact of genetic background on (A) Left kidney weight (g) among nondiabetic and diabetic mice. *P < 0.05 (*n* = 3–17 mice/group). (B) Urine cystatin C to creatinine ratio (CCR; Cystatin C (*μ*g/mL)/Creatinine (*μ*g/mL)) and (C) urine nephrin to creatinine ratio among mice carrying the Akita mutation. *P < 0.05 for (*n* = 3–4 mice/group). For each graph, the maternal strain is indicated on the horizontal axis and strains are in rank order based on the average level observed in diabetic mice. To facilitate comparisons among strains, these data are presented in alphabetical strain order in Figure 7‐4A, 4B, and 4C at the end of the manuscript.

Tissue weights were also assessed as additional reflections of diabetic complications. Absolute kidney weights were significantly greater for most diabetic mice compared to nondiabetic controls (Fig. 4A). Increased kidney weight is one feature of DN often seen in type 1 diabetic humans (Mogensen and Andersen 1973; Bogdanovic 2008) and is associated with hyperfiltration, a predecessor to microalbuminuria. Heart to tibia ratios, a measure of heart hypertrophy and heart failure, showed reduced values for diabetic as compared to nondiabetic mice (data not shown).

### Urinary cystatin C and nephrin levels are elevated in diabetic mice

Cystatin C is a small molecular weight protein with potent inhibitory activity toward lysosomal proteinases and is a marker of tubulus damage (Tan et al. 2002; Kim et al. 2013). In humans, cystatin C levels increase with diabetes, reflecting increased glomerular filtration rates (GFR) during early renal function decline (Tan et al. 2002; Perkins and Krolewski 2009; Vlasakova et al. 2014). Cystatin C was normalized with respect to urine creatinine levels (CCR) in mouse urines in order to evaluate kidney damage due to hyperglycemia.

A main finding here is that CCR levels showed robust increases due to diabetes for most of the strains, increasing 10‐fold for several strains (Fig. 4B). CCR levels were strongly correlated with ACR (*P* = 0.0286). CCR also correlated significantly with plasma TG (*P* = 0.0043) and urine volume (*P* = 0.0455). Thus, urinary CCR may serve as a robust biomarker for kidney damage in mice as is seen in humans.

Nephrin is a transmembrane protein on podocytes located in conjunction with the slit diaphragm. Urinary nephrin is a biomarker specific for podocytes as evidenced by its appearance in urine of diabetic patients but not healthy individuals (Patari et al. 2003). Nephrinuria is seen in FVB/N Akita mice, appearing prior to albuminuria onset (Chang et al. 2012). For strains studied here, enhancement of nephrin was seen among several strains of diabetic mice, but significance was reached for only two strains, BUB/Bn and SJL (Fig. 4C).

### Moderate kidney pathology among Akita mouse strains

**Figure 5.**
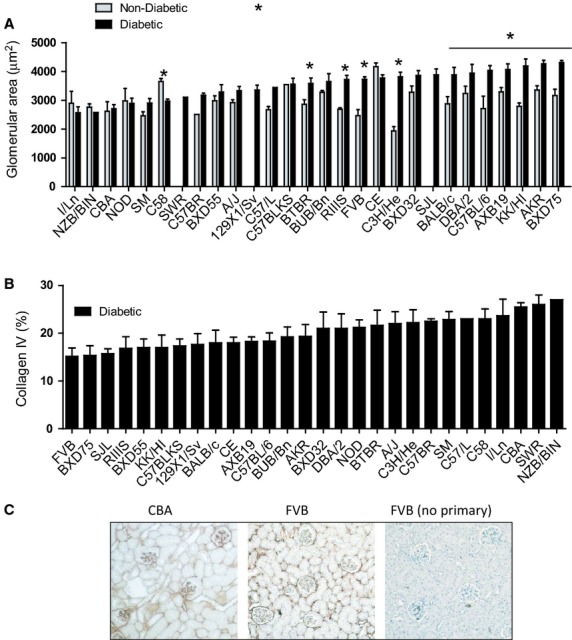
Impact of genetic background on diabetic kidney pathology as measured by (A) Glomerular area (*μ*m^2^) and (B) Percent glomerular area staining for collagen IV. Genetic background variation is determined by the maternal strain, indicated on the horizontal axis. Strains are in rank order based on the average level observed in diabetic mice. (C) Example of collagen IV immunostaining for two mouse strains, CBA and FVB, with one control shown lacking treatment with primary antibody. To facilitate comparisons among strains, these data are presented in alphabetical strain order in Figure 7‐5A, and 5B at the end of the manuscript.

A primary marker of kidney damage and dysfunction is glomerular hypertrophy. Among the nondiabetic strains, areas ranged from 1931 to 4163 *μ*m^2^ (Fig. 5A). With diabetes, six strains showed no change while most of the remaining strains showed modest increases of ~25% and C3H/HeJ showed a 2‐fold increase. Thus, there appear to be genetic factors contributing to glomerular area in both nondiabetic and diabetic mice. Glomerular hypertrophy did not correlate with ACR or with kidney weight, another trait associated with glomerular hypertrophy. In addition, based on visual inspection of H&E stained kidney sections, no major changes in gross structure of the tubulointerstitium were seen for any of the strains.

Since changes in glomerular areas were relatively small, we decided not to pursue formal studies of mesangial expansion (e.g. Periodic acid‐Schiff) but did assess levels of the most abundant glomerular matrix protein, collagen IV (Fig. 5B and C). This protein is a main constituent of basement membranes of glomeruli and tubules and accumulation of type IV collagen is associated with mesangial expansion and tubulointerstitial and glomerular injury in humans (Okonogi et al. 2001). There were two findings from morphological analyses of glomerular collagen IV immunostaining. First, glomerular collagen IV levels were comparable between nondiabetic and diabetic mice within each strain (Fig. 5B and illustrated in Fig. 5C; nondiabetic values not shown). Second, type IV collagen immunostaining varied nearly 2‐fold across the strains and was significantly correlated with ACR values (*P* = 0.020). Thus, collagen IV may provide a predictive trait for albuminuria.

**Figure 6.**
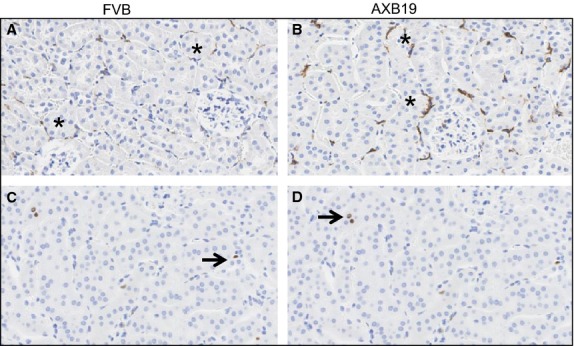
Genetic background does not result in marked changes to tubulointerstitium. Representative tubulointerstitial areas stained with markers for macrophages (F4/80) (A,B) and cellular proliferation (Ki67) (C,D) for FVB and AxB19 mouse strains. Overall tubulointerstitial architecture shows lack of dilation and associated proliferation of tubular epithelial cells. FVB mice show only occasional positivity for macrophages. Although immunostaining for macrophages is somewhat more frequent for AXB19, across six strains examined (AXB19, A/J, KK/HI, C57BL/6, C3H/He, FVB), there was no correlation between the numbers of macrophages and ACR values (data not shown). Thus, macrophage number is unlikely to contribute to diabetic pathology among these mice.

We also examined the tubulointerstitium architecture for several mouse strains with high and low ACR values (Fig. 6). Significant tubular changes were not seen. No signs of tubular dilation, extensive tubular proliferation of epithelial cells, or macrophage invasion were found. Thus, diabetes under conditions rendered here did not provoke extensive interstitial tubule pathology.

**Figure 7.**
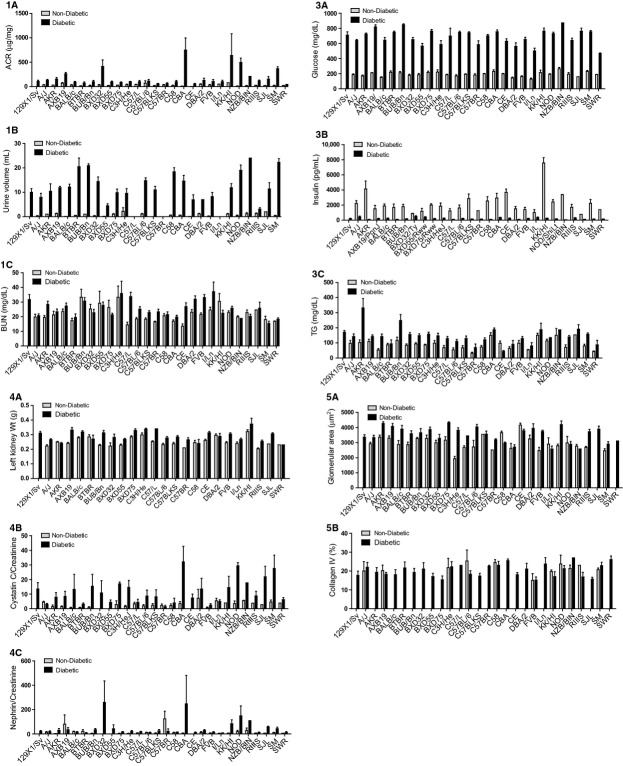
Albuminuria (1A), polyuria (1B), blood urea nitrogen (1C), plasma glucose (3A), plasma insulin (3B), plasma triglycerides (3C), left kidney weight (4A), urine cystatin‐C to creatinine ratio (CCR) (4B), urine nephrin to creatinine ratio (4C), glomerular area (5A), and percent glomerular area staining for collagen IV (5B) for diabetic and nondiabetic control mice across a panel of 28 F1 mouse strains. Panel labels show correspondence with panels in Figures 1, 3–5. For each phenotype, strains are shown in alphabetical order by strain in order to facilitate trait‐trait comparisons across strains. Gray bars show control mice and black bars show diabetic mice. Error bars indicate SEM.

## Discussion

The overall goal of this study was to use the *Ins2* Akita mutation to develop new mouse models of DN and to identify genetically diverse mouse strains with which to identify genes and gene pathways controlling DN. The Akita mutation had been introgressed into five inbred mouse lines with DBA/2 mice showing the greatest response in terms of albuminuria and glomerulopathy (Brosius et al. 2009; Gurley et al. 2010; Yu et al. 2012). Thus, we used DBA/2.Akita mice as donors in breeding the Akita gene onto 28 inbred mouse strains, generating F1 hybrids carrying the Akita mutation as well as euglycemic littermates. We assessed parameters associated with DN. We found that traits associated with kidney structure and function, and plasma lipid levels, varied widely among the strains. A primary finding was the extensive and significant strain variation in ACR values seen with diabetes ranging from no change to macroalbuminuria. ACR did not correlate with glomerular hypertrophy but did correlate significantly with urine cystatin C‐to‐creatinine levels supporting clinical reports using this protein as an early biomarker for kidney dysfunction (Macisaac et al. 2007; Perkins and Krolewski 2009).

In humans, albuminuria is an important indicator of nephropathological abnormities of DN (Fioretto et al. 1992, 2008; Badal and Danesh 2014). For mouse models of DN, an increase in albuminuria of >10‐fold for diabetic as compared with euglycemic controls is one criterion of the National Institutes of Health sponsored AMDCC (www.mmpc.org) (Brosius et al. 2009). Among the 28 strains studied here, most showed 2‐ to 4‐fold increases in ACR with diabetes. These findings agree with other background strains commonly used to study murine DN (Breyer et al. 2008; Gurley et al. 2010). However, three strains surpassed this criterion (12‐ to 16‐fold) (NZB/BIN, SM, BXD32/Ty) and two strains showed robust relative increases in ACRs of 50‐fold (NOD/ShiLt) and 83‐fold (CBA). Another strain, KK/Hl studied by others for DN traits (Shike et al. 2005; Aoki et al. 2012) approached the AMDCC cut‐off (8‐fold). Thus, this survey has identified new murine models in which to study structural features causative for elevated ACR levels. Moreover, genetic crosses between high and low ACR responders can be used to map key genes and gene pathways causative for changes in kidney function that are related to DN.

Information as to the molecular mediators of early kidney disease leading to onset of clinical albuminuria is not known. One recent report suggests that podocyte loss due to ROS‐induced apoptosis may be such a mechanism (Susztak et al. 2006). Interestingly, podocyte apoptosis was initiated by the onset of hyperglycemia in humans and mice (Steffes et al. 2001). In the mouse, podocyte loss remained stable following an initial loss of podocytes with onset of HG suggesting that this mechanism alone is not sufficient to provoke severe pathology in this species.

Although robust changes in albuminuria were seen for some of the strains, none exhibited severe kidney pathology. The largest increase in glomerular area due to diabetes, a trait used to access glomerular hypertrophy, was exhibited by C3H/He for which a 2‐fold increase in glomerular expansion was seen. However, changes in ACR for this strain were small (Fig. 1). In fact, no correlation was seen between ACR and glomerular hypertrophy across the 28 strains suggesting that mechanisms other than mesangial expansion contributed to albuminuria. Although not quantified among these strains, substructural features of renal damage including glomerular basement membrane expansion and/or loss of podocytes could have contributed to extensive albuminuria in some cases. In addition, there was no evidence of tubular injury based on light microscope histopathological analysis of kidneys.

Features of mild kidney pathologies were noted among the mouse strains. For instance, collagen IV accumulation varied 2‐fold among Akita strains and correlated significantly and positively with ACR levels. Nephropathological abnormalities of DN in humans include accumulation of collagen IV, the most abundant extracellular matrix component in the glomerulus (Zent et al. 2006). For mice, excessive fibrillar collagen deposition is seen particularly in mice deficient in integrin *α*1, a collagen IV receptor and negative regulator of glomerulosclerosis (Yu et al. 2012) supporting a role for this matrix protein in promoting renal damage. Of note, collagen IV levels did not correlate with glomerular area suggesting that collagen IV was unlikely to be responsible for glomerular hypertrophy as seen among these strains.

Another feature seen for T1D individuals with risk for DN are enlarged kidneys (Bogdanovic 2008). We also observed significant increases in kidney weight relative to body weight among some, but not all, mouse strains. These results, coupled with the variable outcomes seen for ACR, collagen IV deposition and urine CCR among the strains support reports of the “pathological diversity” of DN within human populations (Valk et al. 2011).

Cystatin C is a small “housekeeping” protein (13,250 Daltons) synthesized by all nucleated cells. Cystatin C is reabsorbed the tubulus cells and rapidly degraded. But with tubulus dysfunction, reabsorption is impaired and cystatin C is eliminated with urine. Thus, cystatin C has been used as a function of tubulus dysfunction and hyperfiltration (Tan et al. 2002; Kim et al. 2013). Among the 28 mouse strains, CCR levels varied 3‐fold among Akita mice and levels correlated significantly and positively with ACR. These data are in agreement with human studies and suggest early changes in hyperfiltration occur in many of these mouse strains. The mouse strains identified here may provide important tools for examination of early modifications of interstitial tubule structure and function with diabetes.

Given the genetic diversity available among inbred mouse strains, we were somewhat surprised in the mild pathology observed in this study, although lack of severe pathology has been noted for several mouse strains previously (Breyer et al. 2008). It is now known that mice are able to develop advanced features of human diabetic nephropathy under conditions for which hyperglycemia is induced on top of genetically modified gene deficiencies. For instance, several groups have demonstrated that loss of eNOS (*Nos3*) results in robust albuminuria and glomerulosclerosis as well as reduced GFR although the extent of changes are background strain dependent (eNOS deficient; Breyer et al. 2008; Zhao et al. 2006). Other cases have been reviewed (Breyer et al. 2008; Brosius et al. 2009). Of note are mice lacking brandykinin studied on the C57BL/6 background (Kakoki et al. 2010), OVE26 mice on the FVB background (Xu et al. 2010) and integrin a1 deficient mice on the BALB/c background (Yu et al. 2012). With introgression of the Akita mutation, all these strains exhibit features of more advanced DN including hypertension, albuminuria, mesangial matrix expansion, and podocyte loss. Thus, our study identifies additional genetic backgrounds that may provide further insight to DN following the incorporation of these gene deficiencies.

More extensive kidney pathology has been seen for type 2 mouse models. Kidney pathology is much worse for eNOS deficient mice when crossed into diabetic and obese leptin deficient strains than wild‐type strains (Zhao et al. 2006; Alpers and Hudkins 2011). An argument can be made that altering plasma lipid levels contributes to worsening DN. For instance, BALB/c mice made diabetic following STZ treatment show an enhancement of glomerulosclerosis when also deficient for the LDL receptor, presumably due to the extensive hyperlipidemia (Wen et al. 2002). Also, proteomic analysis of plasma samples taken from a cohort of type 1 diabetic patients versus matched euglycemic individuals showed that levels of apolipoprotein A1 and C1 as well as 16 other proteins were associated with extent of albuminuria (Nielsen et al. 2010). Finally, among our 28 diabetic strains, levels of plasma triglyceride were significantly and positively correlated with urine volumes and CCR levels. Further work to alter plasma lipid levels among these 28 Akita mouse strains may be worthwhile to test the hypothesis that high fat feeding would enhance DN among these strains.

In summary, this study contributes significantly to broadening our knowledge concerning genetic variations among mouse strains for clinical traits relevant to DN. In particular, we identified multiple strains with robust increases in ACR. Elevated ACR levels are associated with a marked increase in risk of progression to overt proteinuria and eventual ESRD (Caramori et al. 2000; Drummond and Mauer 2002; Mauer and Drummond 2002). However, the course of renal dysfunction and pathological change differ markedly among individuals (Mauer and Drummond 2002; Skupien et al. 2014). Thus, it may be useful to transfer genes potentially causative for DN onto mouse strains exhibiting robust changes in ACR for a better presentation of DN comparable to humans.

## Acknowledgments

We would like to thank Calvin Pan (Division of Cardiology, Department of Medicine, David Geffen School of Medicine, UCLA) for performing correlations studies supporting Figure 2.

## Conflict of Interest

None declared.
